# Effects of Continuous or End-of-Day Far-Red Light on Tomato Plant Growth, Morphology, Light Absorption, and Fruit Production

**DOI:** 10.3389/fpls.2019.00322

**Published:** 2019-03-28

**Authors:** Pavlos Kalaitzoglou, Wim van Ieperen, Jeremy Harbinson, Maarten van der Meer, Stavros Martinakos, Kees Weerheim, Celine C. S. Nicole, Leo F. M. Marcelis

**Affiliations:** ^1^Horticulture and Product Physiology Group, Wageningen University & Research, Wageningen, Netherlands; ^2^Signify, Eindhoven, Netherlands

**Keywords:** far-red, LED, light absorption, photomorphogenesis, shade avoidance, tomato

## Abstract

Shading by sunlit leaves causes a low red (R) to far-red (FR) ratio that results in a low phytochrome stationary state (PSS). A low PSS induces an array of shade avoidance responses that influence plant architecture and development. It has often been suggested that this architectural response is advantageous for plant growth due to its positive effect on light interception. In contrast to sunlight, artificial light sources such as LEDs often lack FR, resulting in a PSS value higher than solar light (∼0.70). The aim of this study was to investigate how PSS values higher than solar radiation influence the growth and development of tomato plants. Additionally, we investigated whether a short period of FR at the end of the day (EOD-FR) could counteract any potentially negative effects caused by a lack of FR during the day. Tomato plants were grown at four PSS levels (0.70, 0.73, 0.80, and 0.88), or with a 15-min end-of-day far-red (EOD-FR) application (PSS 0.10). Photosynthetic Active Radiation (PAR; 150 μmol m^-2^ s^-1^) was supplied using red and blue (95/5%) LEDs. In an additional experiment, the same treatments were applied to plants receiving supplementary low-intensity solar light. Increasing PSS above solar PSS resulted in increased plant height. Leaf area and plant dry mass were lower in the treatments completely lacking FR than treatments with FR. EOD-FR-treated plants responded almost similarly to plants grown without FR, except for plant height, which was increased. Simulations with a 3D-model for light absorption revealed that the increase in dry mass was mainly related to an increase in light absorption due to a higher total leaf area. Increased petiole angle and internode length had a negative influence on total light absorption. Additionally, the treatments without FR and the EOD-FR showed strongly reduced fruit production due to reduced fruit growth associated with reduced source strength and delayed flowering. We conclude that growing tomato plants under artificial light without FR during the light period causes a range of inverse shade avoidance responses, which result in reduced plant source strength and reduced fruit production, which cannot be compensated by a simple EOD-FR treatment.

## Introduction

Plants that normally grow in unshaded or lightly shaded habitats can distinguish differences in the proximity of other plants through alterations in the spectral intensity of light (Casal, 2013). These plants can detect such differences by the ratio of red to far-red light (R:FR), which is detected by a family of plant photoreceptors, the phytochromes (Casal, 2000). Phytochromes have active (Pfr) and inactive forms (Pr) (Chen and Chory, 2011). The ratio of the active Pfr to the total P (Pfr+Pr) is defined as the phytochrome photostationary state (PSS) (Sager et al., 1988). A low R:FR ratio leads to a low PSS, and results in a series of shade avoidance syndrome (SAS) responses. Low R:FR induced SAS responses occur at a plant level and influence the whole plant morphology, including increased stem length and assimilate partitioning toward the stem (Ballare et al., 1991; Smith and Whitelam, 1997; Cole et al., 2011). A low R:FR increases apical dominance and reduces basal branching (Leduc et al., 2014). At the leaf level, a low R:FR increases petiole and leaf length, decreases the leaf mass per leaf area (LMA), and reduces both the leaf chlorophyll content and the chlorophyll *a*:*b* ratio (Smith and Whitelam, 1997; Evans and Poorter, 2001; Sasidharan et al., 2010). A low R:FR also affects plant development by reducing the time to flowering in *Arabidopsis thaliana*, resulting in earlier seed production (Smith and Whitelam, 1997; Dorn et al., 2000). However, the effects of R:FR on fruit formation in fruit producing crops such as tomatoes have been little studied. Additionally, there are few studies on the effects of R:FR using dose-response curves for the quantitative analysis of the effects of R:FR on the morphology and flowering of fruit producing crops.

A short-term, end-of-day (EOD) exposure of plants to a low R:FR ratio can already result in responses typical for continuous low R:FR, such as increased internode, stem, and petiole lengths (López-Juez et al., 1990; Xiong et al., 2002; Chia and Kubota, 2010; Yang et al., 2012). Other reported responses to EOD-FR are increased stem dry weight, reduced leaf chlorophyll content, and reduced leaf area (Graham and Decoteau, 1997; Lund et al., 2007). The responses of plants to continuous FR and short-term EOD-FR applications have been investigated separately, and a systematic comparison of effects of continuous FR and EOD-FR is lacking.

As R:FR affects plant architecture it also changes the total light absorption by the plant, as well as the distribution of absorbed light over the whole plant (Smith and Whitelam, 1997). Heraut-Bron et al. (2001) suggested that total light interception by white clover plants was increased by increased petiole elongation under low R:FR. In their study neither the area of individual leaves, nor the optical properties of the leaves, were affected by R:FR. Other authors have also assumed that the increased elongation of plants under low R:FR resulted in an increase in light interception (Dudley and Schmitt, 1996). However, the consequences of changes in morphology due to changing R:FR ratios upon plant light absorption are hard to quantify, and this remains a major gap in our understanding of photomorphogenic responses. Today, it is possible to quantify the effects of plant architecture on plant and crop light absorption and photosynthesis due to the introduction of functional-structural plant models (FSPMs). FSPMs are tools that use an explicit 3D plant architecture combined with organ-specific optical properties (Vos et al., 2010; Sarlikioti et al., 2011a). This combination allows the simulation of the interaction between plants and the three-dimensional distribution of light (Vos et al., 2010; Bongers et al., 2014).

Alongside its impacts on plant morphology and development, FR may also affect the photosynthetic performance of the leaf. Due to the Emerson enhancement effect, the combination of R and FR light may result in a higher photosynthetic rate compared to applying both wavelengths independently (Emerson et al., 1957; Pettai et al., 2005). There are, yet, few studies on the effects of FR on photosynthesis of crops grown in greenhouses with LED lighting.

Light-emitting diodes (LEDs) are increasingly used in greenhouses, vertical farming and growth cabinets, with or without natural daylight (Hogewoning et al., 2007; Demotes-Mainard et al., 2016). LEDs are characterized by relatively narrow-band spectra that does not resemble natural daylight, which is continuous in the PAR region (400–700 nm). Additionally, the LEDs used in modern horticulture emit minimal to zero light in the region of FR (710–850 nm), resulting in R:FR ratios that are higher than those of natural daylight. The effects of additional FR or a less energy consuming EOD-FR treatment on the morphology and productivity of fruit producing crops grown under horticultural LEDs still need to be investigated. For such research, investigating the effect of complementing the FR-enriched LEDs with natural daylight is necessary, to avoid potentially unwanted effects due to the absolute lack of certain wavelengths (other than red and blue).

The main aim of this study was to investigate the effects of the higher than sunlight R:FR ratio of artificial light supplied by a combination of red, blue, and far-red LEDs on different morphological parameters and how changes in these parameters affect the total light absorption, and consequently, the growth of tomato plants. The secondary objectives were to (1) identify whether there are differences in plant responses to continuous or end-of-day presence of FR, and (2) investigate whether the effects of FR on plant morphology depend on the presence of broadband background radiation.

## Materials and Methods

Two experiments (EXP1 and EXP2) were performed with tomato plants in the glasshouses of Wageningen University. EXP1 started on the 3rd of February 2015 and EXP2 started on the 13th of March 2015. Each experiment lasted initially for 4 weeks, but EXP2 was continued for 12 additional weeks to allow plants to form fruit trusses for yield measurements. In EXP1, five FR-light treatments were compared under conditions where solar light was almost completely blocked, while in EXP2 the same treatments were compared under conditions where solar light was allowed to enter the greenhouse.

### Plant Material and Growth Conditions

Tomato seeds (*Solanum lycopersicum* “Komeett”) were sown in a mixture (50–50%) of perlite and potting soil (*Arabidopsis* soil, Horticoop, Netherlands) in a greenhouse. After the second true leaf had appeared (15 day in EXP1 and 17 days in EXP2), the seedlings were transplanted into pots containing ∼6300 cm^3^ of quartz sand. Every pot had a dripper from which a nutrient solution (EC 2.0, pH 5.5) was provided (Supplementary Table S1). Flowers in EXP 2 were pollinated by mechanical vibration three times a week.

The greenhouse was divided in 15 compartments of 1.5 m × 4.5 m separated by white plastic screens. There were two rows of 10 plants per compartment, with 40 cm distance between plants within the same row and 30 cm between plants in different rows. The distance between a row and its proximal plastic screen was 60 cm. The first and last plants on each row served as border plants. During EXP1, the black-out screen of the greenhouse (Ludvig Svensson SL99, which blocks 98% of the solar daylight), was kept closed so that plants received negligible levels of solar light (solar light contributed less than 1% of total light). The black-out screen, was placed at the level of the rain gutter (6 m from the floor and 5.5 m from the pots). During EXP2, the same black-out screen was opened after sunrise (06.20 AM solar time) and closed before sunset (03:20 PM solar time) to avoid the temporary increase in the R:FR ratio of daylight at twilight. The shading screen of the greenhouse (Ludvig Svensson SLS10, 81% transmission) was closed for the entire duration of EXP2 to diffuse the solar light. The shading screen, designed to reflect part of the solar light during the summer months, was placed a few centimeters below the black-out screen. In both experiments, the day/night temperature in was 23/18°C, 56–64% relative humidity, and 394–437 ppm CO_2_ concentration (Supplementary Table S2). Temperatures were measured with two thermocouples (K type) per experimental plot, covered with aluminum shields to protect them from direct light.

From transplanting, plants in all treatments were illuminated by a mixture of blue (5%) and red (95%) LEDs (Philips GreenPower LED top lighting module DR/B LB), with dominant wavelengths of 450 and 638 nm, respectively (Supplementary Figure S1). At the apex height, all plants received ≈150 μmol m^-2^ s^-1^ PAR for 16 h per day from LEDs in both experiments, and in EXP2 there was additional diffuse solar radiation with a daily maximum of around ≈50 μmol m^-2^ s^-1^ PAR around noon (Table 1). FR light was provided at five different intensities by LEDs (Philips GreenPower LED FR 150) with dominant wavelengths of 730 (Table 1 and Supplementary Figure S1). In four treatments, the FR lamps were on at the same time cycle as the red/blue lamps, creating four different PSS values during the day (Table 1). The fifth treatment was an end-of-day (EOD) treatment, where the FR lamps were on for only 15 min after the end of the photoperiod. The spectral intensity was measured in every plot using a spectroradiometer (USB2000 spectrometer, Ocean Optics, Duiven, Netherlands). The PSS values were calculated following the methods of Sager et al. (1988). The presence of solar light in EXP2 reduced the PSS value of the EOD treatment by 1%, compared to EXP1. The height of the LED arrays was adjusted every 2 days to ensure constant irradiance throughout the experiment. Irradiance was measured every 2 days using a quantum sensor (LI-190, LiCor Inc., Lincoln, NE, United States), and verified with the spectroradiometer. To ensure the same intensity of solar light in all treatments in EXP2, the shading for the LED modules was made to be identical for all LED light treatments by the addition of dummy FR LED modules.

**Table 1:** LED and solar light intensity (PAR and FR) and calculated PSS values for the five treatments without solar light (EXP1) or with solar light (EXP2).

	EXP1 (LED)					EXP2 (LED + solar)				
Treatment	PSS0.70	PSS0.73	PSS0.80	PSS0.88	EOD	PSS0.70	PSS0.73	PSS0.80	PSS0.87	EOD
PSS	0.70	0.73	0.80	0.88	0.88/0.1^∗^	0.70	0.73	0.80	0.87	0.87/0.1^∗^
PAR LED (μmol m^-2^ s^-1^)	149 ± 0.1	148 ± 0.4	149 ± 0.1	149 ± 0.4	149 ± 0.2	159 ± 2	152 ± 2	146 ± 3	149 ± 6	155 ± 5
PAR solar (μmol m^-2^ s^-1^)	0	0	0	0	0	40 ± 2	39 ± 6	38 ± 3	43 ± 3	50 ± 2
PAR solar fraction (%)	0	0	0	0	0	9.3	9.2	9.3	10.6	11.3
FR solar (μmol m^-2^ s^-1^)	0	0	0	0	0	11 ± 1	11 ± 1	10 ± 1	11 ± 1	13 ± 2
FR LED (μmol m^-2^ s^-1^)	154 ± 0.8	119 ± 1.1	54 ± 0.7	0	17 ± 0.4	154 ± 1	113 ± 2	49 ± 2	0	17 ± 0.5

*The values are the means of three measurements at plant height per plot (three plots per treatment). The standard deviation was calculated based on the averages per plot. Data was acquired by spectroradiometer on the 12th of February (9:20–11:20 AM solar time) and on the 17th of March (9:20–10:20 AM solar time) for EXP 1 and 2, respectively. In EXP2, PSS values are based on the combination of lamp and solar light (the PSS values of lamp light were equal to those of EXP1). EOD is end-of-day lighting, meaning the FR lamps were on for 15 min just after the end of the 16-h photoperiod. ^∗^The first value refers to the period that the red/blue LEDs operated, the second value when only the FR LEDs operated*.

### Growth and Morphology Analysis

Four weeks after transplanting, petiole angles (upper angle between petiole and stem) were measured at midday using a protractor (Graham and Decoteau, 1997). The following day, plants were harvested and dissected into different parts: leaves plus petioles, cotyledons, hypocotyls, internodes, flowers, roots, and apex. After dissection, each internode, petiole and hypocotyl length, as well as leaf length and width (the distance between the two most extended leaflets of the composite leaf), were measured with a ruler. Each leaf and cotyledon area were measured with a leaf area meter (LI-3100C Area Meter, LiCor Inc., Lincoln, NE, United States). The total plant leaf area included the cotyledons. The plant parts were oven-dried at 70°C for 16 h, followed by 22 h at 105°C. In EXP2, the number of red and green fruits, and their fresh weight, were measured 16 weeks after transplantation (no destructive measurements were done). No fruits were harvested before that date.

### Leaf Gas Exchange Measurements

In EXP2, 4 weeks after transplanting gas exchange was measured on leaf 4 or 5, using an LI-6400XT portable photosynthesis system (LiCor Inc., Lincoln, NE, United States) with a transparent leaf chamber. During the measurements, solar light was blocked by the black-out screens, which were temporarily closed to allow for a precise 150 μmol m^-2^ s^-1^ light intensity inside the leaf chamber. This was confirmed using a spectroradiometer. Chamber air temperature was set at 23°C, relative humidity at 65%, CO_2_-concentration at 400 μmol CO_2_ (mol air)^-1^, and the air flow rate was 500 μmol s^-1^. Gas exchange was measured continuously for 15 min with 10 s intervals; the average of the data of the last minute were used for analysis.

### Leaf Optical Properties and Pigment Content

Light absorbance on the adaxial side of leaves was determined by measuring the transmittance and reflectance of leaf disks (without veins) of leaf 4 and 8 with two integrating spheres (Hogewoning et al., 2010). Leaf absorbance for PAR was calculated using the spectral distribution of the LED lights used in the experiment.

Leaf disks used for light absorbance were stored at -80°C, and then used to measure total chlorophyll and carotenoid content after extraction in *N*, *N*-dimethylformamide. The absorbance of the extract was measured at 663.8, 646.8, and 480 nm using a Cary 4000 spectrophotometer (Varian Instruments, Walnut Creek, CA, United States), and the concentration of the chlorophylls and carotenoids calculated according to Wellburn (1994).

### Modeling Light Absorption

A 3D structural plant model was constructed within GroIMP (Growth Grammar-related Interactive Modeling Platform) (Hemmerling et al., 2008). The model includes (i) a static representation of the three-dimensional (3D) architecture of the tomato plants as well as the physical layout of the plots in the greenhouse, and (ii) a radiation model to simulate light capture of individual leaves.

In the model the architecture of the plants was re-constructed based on the petiole angle, petiole length, leaf area, leaf length, internode length, and the leaf optical properties, as measured for each treatment at the final harvest of EXP1 (Supplementary Figure S2). Phyllotactic angles of the leaves were assumed to be the golden angle of 137.5°. Rachis curvature was set at 102, 103, and 121° for, respectively, leaves 1–3, 4–6, and 7–11 (Supplementary Figure S3). These curvature angles were based on pictures taken at final harvest and were considered the same for all treatments. Leaflet curvature could not be estimated from pictures; therefore, each simulation was run with three leaflet curvature scenarios with angles set to 0, 15, and 30° (Supplementary Figure S4). Leaflet shape was estimated from pictures taken of the leaves of the same variety in a different greenhouse experiment, following the approach of Evers et al. (2006). The leaflet shape parameter values L_m_ (the distance of the point of maximum margin–midrib distance to the blade tip as a fraction of the final length) and c (a curvature coefficient) were both estimated to be 0.7 for all treatments. The base of each plant was oriented randomly between 0 and 360°. As this introduces some variation in the outcome of the simulation, each scenario was simulated five times, with 20 plants per simulation (i.e., each scenario mimicked a complete plot). Plant spacing was according to the actual spacing data in the greenhouse.

With respect to the physical layout of the greenhouse, the dimensions and position of heating pipes, concrete floor, gutters and the vertical screens were reconstructed in the model. Reflectivity of heating pipes, concrete floor, and gutters was assumed to be 15%. Reflectivity of the curtains was set at 80%, as measured by the manufacturer (Oerlemans Plastics BV, Netherlands). Reflectivity was assumed to follow a Lambertian distribution. For the curtains, this assumption was checked by measuring the reflectivity distribution, using the methods described by Hemming et al. (2016).

Computation of the light environment was performed using a Monte-Carlo ray tracer embedded in GroIMP (Hemmerling et al., 2008). Light absorption, reflection and transmittance of each individual leaflet were calculated based on the amount of light reaching that leaflet and the leaflet’s optical properties (Buck-Sorlin et al., 2011). Red and blue LED light sources were modeled with associated light distribution and light spectrum as used in the experiment.

The light absorption was simulated for each treatment separately. Subsequently the impact of the response of morphological plant parameters to the far-red light treatments on plant light-absorption was estimated. The simulated light absorption based on the parameter values measured in the PSS 0.88 treatment were used as a reference. For each scenario, the value for one of the parameters (petiole angle, petiole length, leaf area, leaf length, internode length, or leaf optical properties) was changed to that measured for one of the other treatments. In addition to these simulations, the effect of internode length was also simulated for a more extensive canopy of 600 plants. The results of the plants in the center of this more extended canopy were used for further comparison and analysis.

### Statistics

Treatments were arranged in a randomized design where the five light treatments were distributed over 15 plots. Hence, three replicate plots were used per treatment. In both experiments, measurements were performed on four plants per plot for growth and two plants per plot for leaf light absorbance and pigment analysis. In EXP2, four additional plants per plot were used for photosynthesis measurements, and five plants per plot for fruit production measurements. The data from EXP1 and EXP2 were each analyzed by a one-way ANOVA and *post hoc* Fisher’s LSD (*P* < 0.05).

## Results

### Plant Morphology

**Figure 1:**
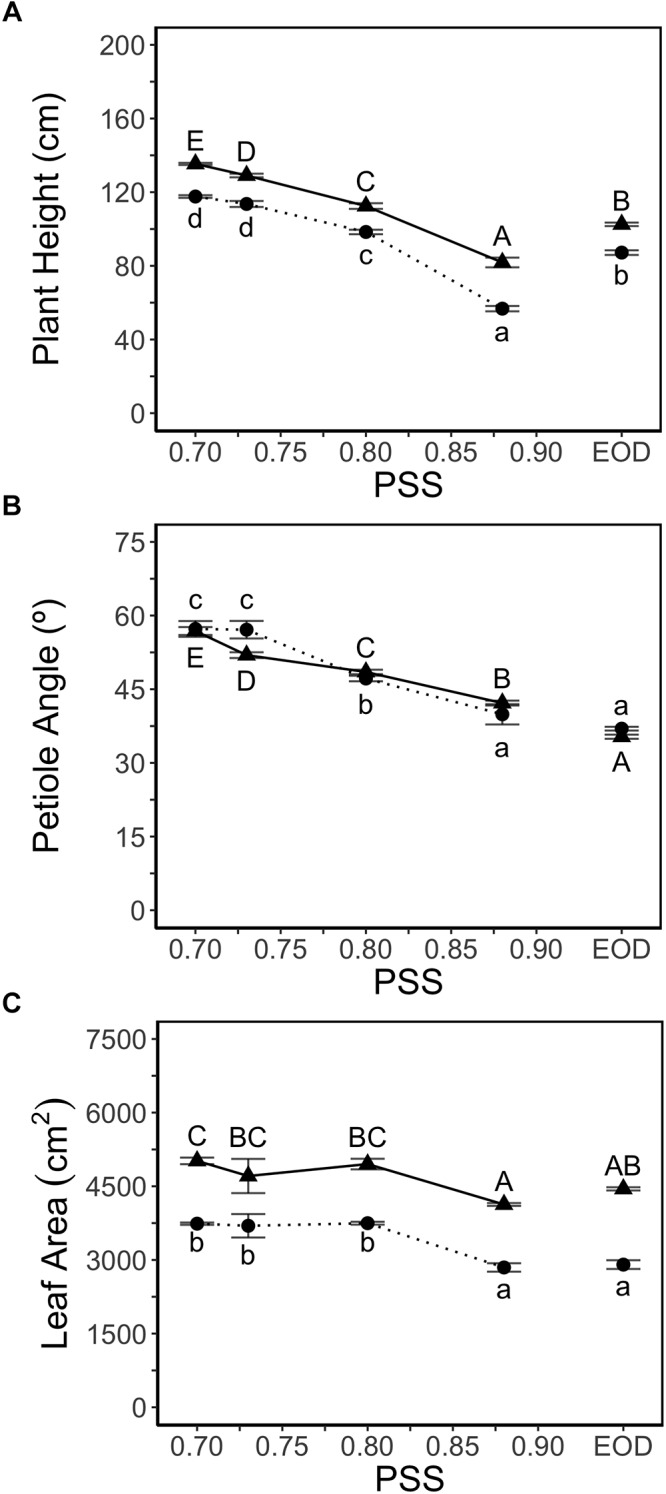
**(A)** Height, **(B)** average petiole angles of all leaves (upper angle between petiole and stem) and, **(C)** leaf area of tomato plants in relation to four levels of PSS and an EOD-FR treatment (EXP1: 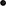, lower case letters; EXP2: 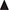, capital letters). Plants had been grown for 4 weeks at the different light treatments. Error bars represent SEM (*n* = 3). Different letters indicate significantly different means (*P* < 0.05).

Plant height increased with decreasing PSS in both experiments (Figure 1A). Plants treated with EOD-FR were taller than plants without FR (highest PSS; Figure 1A). FR treatments did not affect the number of leaves (11 leaves after 4 weeks of growth) in either experiment. Hence, the internode length averaged over all internodes displayed the same trend as plant height (data for hypocotyl and internode length in Supplementary Table S3).

Petiole angle (upper angle between petiole and stem) increased with decreasing PSS in both experiments, resulting in more horizontally oriented leaves at low PSS (Figure 1B). The EOD-FR treatment hardly affected the petiole angle. Decreasing PSS from 0.88 (0.87 in EXP2) to 0.80 increased the total leaf area per plant by approximately 3% (EXP1) and 21% (EXP2) (Figure 1C) but decreasing PSS below 0.80 had no further effect on total leaf area. The EOD-FR treatment did not increase total leaf area, compared with the other treatments without supplemental FR (PSS 0.88; Figure 1C). The area of individual fully grown leaves (Leaf number 2 and 3) showed comparable results to that of total leaf area, and leaf length increased with decreasing PSS in both experiments (Supplementary Table S3). The leaf mass per leaf area (LMA) was lower for the plants treated with additional FR or EOD-FR (Supplementary Table S4).

### Plant Growth

**Figure 2:**
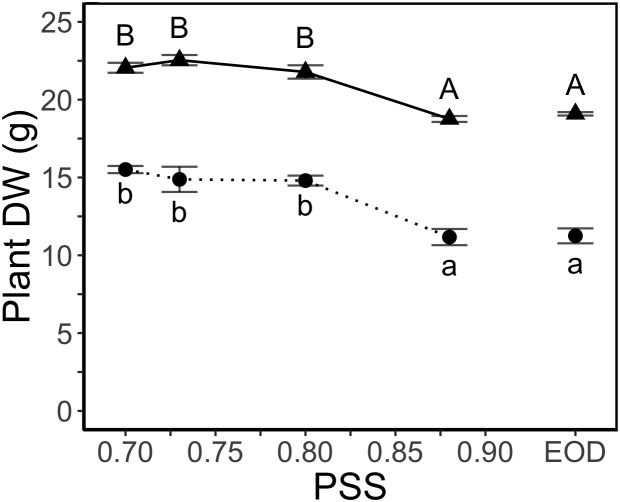
DW of tomato plants in relation to four levels of PSS and an EOD-FR treatment (EXP1: 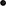, lower case letters; EXP2: 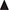, capital letters). Plants had been grown for 4 weeks at the different light treatments. Error bars represent SEM (*n* = 3). Different letters indicate significantly different means (*P* < 0.05).

Decreasing PSS from 0.88 to 0.80 increased the total dry weight per plant (Figure 2) but decreases in PSS below 0.80 had no further effect on total plant dry weight. The EOD-FR treatment did not compensate for the lack of FR (i.e., PSS 0.88) during the daytime (Figure 2).

**Figure 3:**
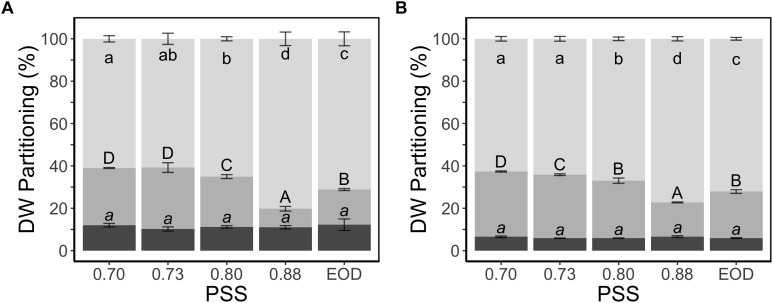
Dry weight partitioning (%) to different plant organs of tomato plants in relation to four levels of PSS and an EOD-FR treatment in EXP1 **(A)** and EXP2 **(B)**. Plants had been grown for 4 weeks at the different light treatments. Error bars represent SEM (*n* = 3). Different letters (lower case for leaves, capitals for stem and lower-case italics for roots) indicate significantly different means (*P* < 0.05).

The fraction of dry weight partitioned to the stem was two times greater for the plants with the lowest PSS than the plants with the highest PSS, and this change in allocation was at the expense of partitioning to the leaves (Figure 3). The EOD-FR treatment increased the fraction of dry weight partitioned to the stem, again at the expense of the leaves. Partitioning to the roots did not show any substantial difference between the treatments.

After 4 weeks of growth, the plants with a PSS of less than 0.88 and those from the EOD-FR treatment had at least two open flower buds (Table 2). In contrast, plants with the highest PSS level had no open flowers. After 4 months, however, there were no significant treatment effects on the total number of fruit trusses formed. In contrast, the total number and fresh weight of fruits per plant, as well as average fruit fresh weight, were significantly higher for the plants with a PSS of less than 0.88 and those from the EOD-FR treatment. The total fruit fresh weight was 59% greater for plants with a PSS of 0.70 than that in plants with a PSS of 0.88 (Table 2). The percentage of fruits that had already turned red increased significantly with decreasing PSS. The number of fruits per truss seemed to increase, although not significantly, with decreasing PSS.

**Table 2:** Formation of flowers, trusses, and fruits of tomato plants in relation to four levels of PSS and an EOD-FR treatment with additional solar light (EXP2).

PSS	0.70	0.73	0.80	0.87	EOD
Nr of open flowers on Week 4	3.0 c	2.3 b	2.2 b	0.0 a	2.1 b
Nr of trusses	7.9 a	8.3 a	7.9 a	7.7 a	7.5 a
Nr of fruits	39.9 c	40.7 c	41.5 c	32.5 a	35.2 b
Nr of fruits per truss	5.1 a	4.9 a	5.2 a	4.3 a	4.7 a
Red fruits (%)	20.1 c	18.0 c	13.2 b	9.4 a	10.4 a
Total fruit FW (g/plant)	2849 d	2934 e	2722 c	1791 a	1992 b
Individual fruit FW (g/fruit)	71.5 c	72.0 c	65.6 b	55.0 a	56.58 a

*Number of open flowers per plant were recorded 4 weeks after start of the treatments, while other fruit data after 4 months: number of fruit trusses and fruits, percentage red fruits, and total fruit weight per plant. Different letters indicate significantly different means (*P* < 0.05)*.

### Leaf Light Absorbance, Net Photosynthesis, and Pigments

**Figure 4:**
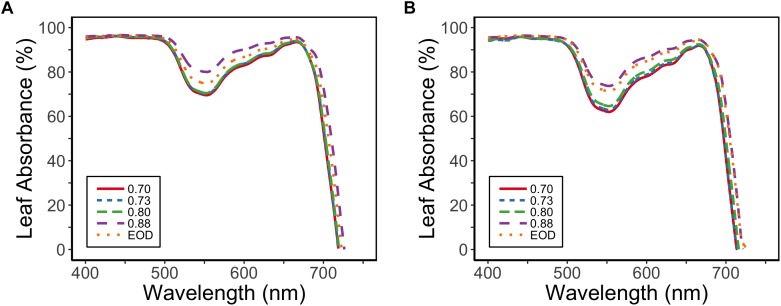
Absorbance spectra for tomato leaves in relation to four levels of PSS and an EOD-FR treatment in EXP1 **(A)** and EXP2 **(B)** (*n* = 2). Plants had been grown for 4 weeks at the different light treatments.

Leaf absorbance for PAR (400–700 nm) was reduced for the plants with lower PSS values; PAR absorbance was 92% at a PSS of 0.88, but was 89% for EOD-FR and 87% for the other PSS treatments (EXP1) (Figure 4). The difference in the absorbed PAR between the treatments was greatest at 550 nm.

The chlorophyll (Chl *a* + Chl *b*) and carotenoid contents per unit leaf area as well as the Chl *a*/Chl *b* ratio of leaf 4, were significantly higher when there was no FR (PSS 0.87–0.88) compared to all the treatments where FR was added during the photoperiod (PSS 0.70–0.80) (Table 3). In EXP1, for the treatment with PSS 0.88 chlorophyll and carotenoid content was also higher than for the EOD-FR, but in EXP2 these differences were not significant.

**Table 3:** Chl *a*, *b* and carotenoid content of leaf disks cut from leaf 4 of tomato plants in relation to four levels of PSS and EOD-FR treatments without solar light (EXP1) or with solar light (EXP2).

	EXP1 (LED)					EXP2 (LED + solar)				
PSS	0.70	0.73	0.80	0.88	EOD	0.70	0.73	0.80	0.87	EOD
Chl *a* (μg cm^-2^)	14.05 a	14.91 b	14.97 b	20.30 d	16.98 c	13.40 a	13.07 a	13.29 a	17.97 b	17.29 b
Chl *b* (μg cm^-2^)	3.93 a	4.10 ab	4.05 ab	5.23 c	4.34 b	3.90 a	3.82 a	3.60 a	4.84 b	4.83 b
Ratio Chl *a*:*b*	3.56 a	3.63 ab	3.69 b	3.89 c	3.92 c	3.43 a	3.42 a	3.69 b	3.70 b	3.58 b
Carotenoid (μg cm^-2^)	2.86 a	2.60 a	2.67 a	4.00 b	3.14 c	2.52 a	2.41 a	2.44 a	3.29 b	3.12 b

*Plants had been grown for 4 weeks at the different light treatments. Different letters indicate significantly different means (*P* < 0.05)*.

Net leaf photosynthesis (measured after 4 weeks of EXP2 for three of the five treatments) had a maximum at a PSS of 0.80 (Table 4). Plants grown with a PSS of 0.70 had a significantly (*P* < 0.05) lower stomatal conductance than plants with a PSS of 0.80 and 0.87 (Table 4).

**Table 4:** Net leaf photosynthesis (A) and stomatal conductance (g_s_) of tomato leaves in relation to three levels of PSS, measured under growth light spectrum (total light intensity ≈160 μmol m^-2^ s^-1^).

PSS	0.70	0.80	0.87
A (μmol CO_2_ m^-2^ s^-1^)	5.34 ab	5.76 b	5.02 a
g_s_ (mol H_2_O m^-2^ s^-1^)	0.07 a	0.11 b	0.10 b

*Plants had been grown for 4 weeks at the different light treatments (EXP2). Different letters indicate significantly different means (*P* < 0.05)*.

### Plant Light Absorption

**Figure 5:**
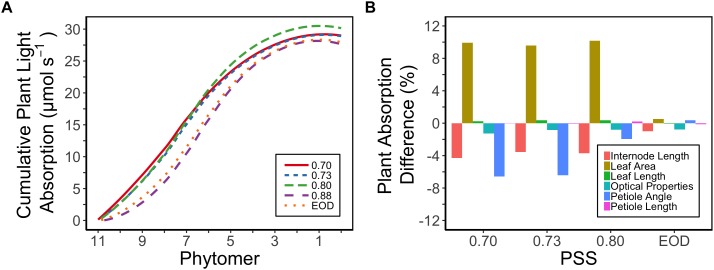
**(A)** Calculated cumulative (starting from the plant top, i.e., Phytomer 11) light absorption of tomato plants per phytomer (leaf + internode + petiole) in relation to four levels of PSS and an EOD-FR treatment in EXP1 (total light intensity of 150 μmol m^-2^ s^-1^). Plants had been grown for 4 weeks at the different light treatments, separated by vertical screens. Calculations were done using a functional-structural plant model based on the average plant morphological and optical parameters of each treatment. The phytomers were counted acropetally where phytomer 1 represents the hypocotyl and two cotyledons. **(B)** Relative impact of the morphological parameters (internode length, petiole angle, leaf area, leaf length, petiole length) and optical properties of the leaves (transmittance, reflectance) on the calculated plant light absorption. Plants with PSS 0.88 were taken as reference. Light absorption of the plants was calculated by replacing the parameter values of plants with PSS 0.88 by parameter values taken from plants with PSS 0.70, 0.73, 0.80, and EOD. After one parameter was changed, the plant absorption was compared to the original absorption of the plants with PSS 0.88.

Plant light absorption was calculated using a functional-structural plant model based on the measured morphology of the plants and the optical properties of the leaves. Tomato plants with a lower PSS in general exhibited a higher total light absorption than plants with a higher PSS or to those grown with EOD-FR (Figure 5A). The plants with a PSS of 0.80 had the highest total light absorption. Plants treated with EOD-FR had a similar total light absorption to plants with the highest PSS (Figure 5A). When only the top six leaves (phytomer 11–6) were considered, the treatment effects on light absorption were stronger than for the whole plant. The top of the plant could also be considered as an approximation of light absorption for plants at a younger stage, when only the first six leaves had been formed.

**Figure 6:**
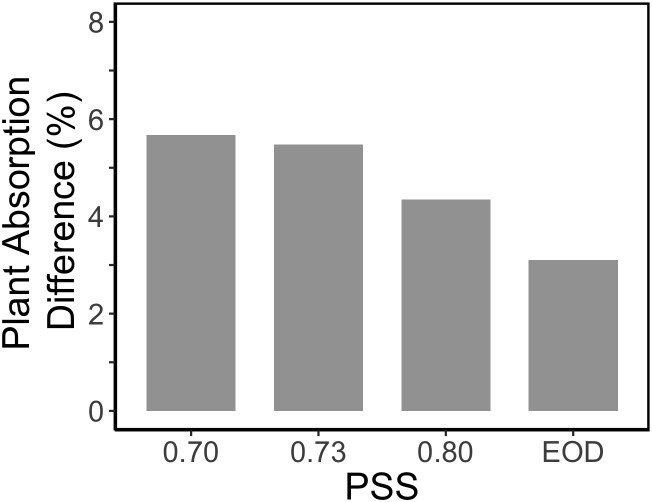
Relative impact of internode length on the calculated light absorption of tomato plants in an extended canopy (no presence of vertical screens). The tomato plants had a default plant architecture of the PSS 0.88 treatment, and only the parameter internode length was changed by taking the internode length values from plants with PSS 0.70, 0.73, 0.80, and EOD.

**Figure 7:**
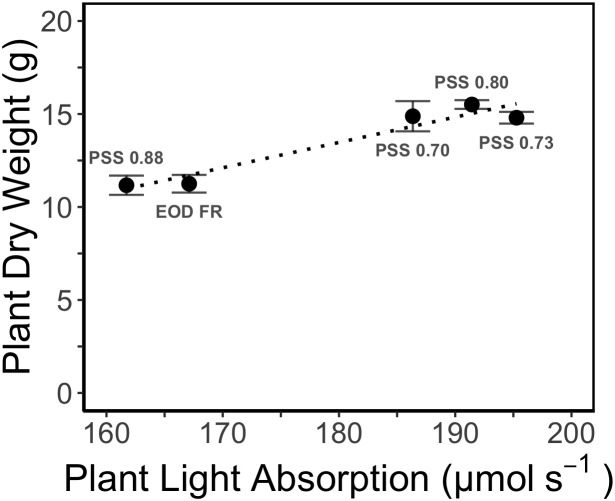
Relation between simulated whole plant light absorption and measured dry weight (DW) of tomato plants grown for 4 weeks under different light treatments in EXP1. The dotted line represents linear regression (*r*^2^ = 0.93, *P* < 0.01). Error bars represent SEM (*n* = 3).

The increased leaf area in treatments with a PSS value lower than 0.88 caused a distinct increase (approximately 10%) in total light absorption of the plants (Figure 5B). Comparing the consequences of FR effects on the different morphological parameters, the FR effects on leaf area had the strongest impact on total plant light absorption (Figure 5B). Changes in petiole and leaf length due to low PSS values had only a minor positive effect on the total light absorption (Figure 5B). In contrast, changes in petiole angle and internode length in plants with a low PSS had a negative effect on the total light absorption. The differences in leaf optical properties due to differences in PSS had only minor effects on total light absorption (Figure 5B). Light absorption by the EOD-FR plants differed less than 1% from that of plants with a PSS of 0.88 (Figure 5B). In the experiment, which was mimicked by the simulation model, each plot of 20 plants was surrounded by vertical plastic sheets. When the calculations by the model were performed for an extensive canopy (without vertical sheets) an increase in internode length increased plant light absorption (Figure 6). Lastly, plant dry weight correlated linearly with whole plant light absorption (*P* < 0.01, *r*^2^ = 0.91; Figure 7).

## Discussion

### Morphology, Light Absorption, and Fruit Production

Many of the previous studies about plant responses to R and FR are associated with the Shade Avoidance Syndrome (SAS). In these studies, usually a high R:FR ratio is compared with a very low R:FR ratio, which occurs in shadow underneath vegetation. An elaborate overview has recently been published in Demotes-Mainard et al. (2016). Most of these studies aim at unraveling physiological mechanisms. Conversely, in many important horticultural systems, the increasing use of LEDs generates R:FR ratios that are significantly larger than the R:FR ratio observed under natural sunlight. This study for the first time systematically investigated the effect of these higher than sunlight R:FR ratios in a dose response manner. Additionally, a first attempt was made to relate the observed morphological changes to whole plant light absorption and observed plant DW.

Plant DW clearly increased with decreasing PSS (increasing FR) (Figure 2). This increase in plant DW was mainly related to differences in light absorption, which in turn were mainly due to differences in total leaf area rather than changes in other morphological and phototropic parameters (petiole, leaf and internode length, petiole angle, optical properties) (Figure 5B). The increasing leaf area with decreasing PSS at above-sunlight R:FR ratios is in contrast with the often-observed inhibitory effect of increasing FR on resource allocation to leaves, while favoring the stem. This is often mentioned as a shade avoidance syndrome hallmark. Additionally, it is important to consider that among various dicotyledonous species, decreases in R:FR ratio can cause both inhibition and stimulation of leaf expansion (Demotes-Mainard et al., 2016; and references herein).

The majority of previous studies on phototropic responses associated with the SAS, report that decreasing PSS results in reduced petiole angles (upper angle between petiole and stem) (Whitelam and Johnson, 1982; Kozuka et al., 2010; Sasidharan et al., 2010). In this study, we observed that decreasing PSS resulted in increased petiole angle (Figure 1B). In contrast to previous studies, in the present study, the effect of higher R:FR ratio than that of the natural sunlight was studied, which may explain these differences in results. Nonetheless, the petiole angle did not have a major effect on the simulated amount of light absorbed as the plants had formed a dense canopy, possibly due to overlapping leaves and mutual shading (Figure 5B).

Surprisingly, the increased internode length at low PSS had a negative effect on the simulated plant light absorption (Figure 5B); in contrast, Sarlikioti et al. (2011b) estimated an increase in total light absorption when internode length increased. This calculated negative effect of internode length on plant light absorption was related to the fact that plants were grown in relatively small experimental compartments (1.5 × 4.5 m). When internode and thus plant height increased, lamp height was also increased to maintain the same light intensity at top of the canopy. The higher the lamps, the more light was lost to the side walls; hence more light was lost to the side walls in the low PSS treatments where internode elongation was stimulated. This is a normal, but usually overlooked, phenomenon found in small compartments where light intensity decreases with distance from lamps even if there are no plants. Nonetheless, ensuring that the distance between lamps and plant-top remains constant is better than maintaining the lamps at same height from the floor. However, this aspect deserves more attention in experiments where plant height is affected by treatments. Based on the extended canopy simulations, we can conclude that the positive effects of FR light on plant mass would have been more substantial if treatments had been applied in large compartments without effects of side walls (Figure 6).

Although calculations of whole-plant light absorption were done only for one plant growth stage (4 weeks after start of treatments), extrapolating the differences observed to earlier plant growth stages can be done via the cumulative light absorption per plant phytomer (Figure 5A). For a plant with only six leaves, the plants with lowest PSS absorbed 63% more light than plants with highest PSS, while the plants only absorbed 16% more light when 11 leaves had been formed. This shows that lower PSS induces an acceleration in plant growth, particularly in the canopies of small young plants where competition for light is still limited.

The positive effect of low PSS on leaf area and light interception probably also contributed to the increased fruit production, as an increase in source strength in plants can stimulate fruit set and growth of individual fruits (Marcelis, 1996; Table 2). Moreover, additional FR accelerated flowering in tomato, which is in line with the effects on flowering time in *A. thaliana* (Halliday et al., 1994; Smith and Whitelam, 1997; Dorn et al., 2000). The accelerated flowering was likely one additional reason for the strong stimulation of fruit production with decreasing PSS (Table 2). Furthermore, the increased fruit production could be due to direct effects of FR light on fruit set, assimilate partitioning, and fruit growth. Effects of FR light on fruit set and assimilate partitioning to fruits are hardly explored yet. This subject need further investigation.

### Leaf Light Absorption, Pigmentation, and Photosynthesis

At the leaf level, the lower PAR absorbance of plants with a lower PSS corresponded well with their lower total chlorophyll concentrations and chl *a*:*b* ratios (Table 3). These results are in accordance with studies on shade acclimation responses (Smith and Whitelam, 1997; Evans and Poorter, 2001). Lower PSS levels also decreased the carotenoid concentration, as determined by Li and Kubota (2009).

Despite the lower leaf PAR absorbance (Figure 4) and lower photosynthetic pigment content (Table 3), tomato leaves grown at an intermediate PSS of 0.80 showed higher leaf photosynthesis rates than those grown at a PSS of 0.87 (without FR; Table 4). Since FR does not contribute to PAR, this effect could have been due to the Emerson enhancement effect (Emerson et al., 1957; Pettai et al., 2005). Surprisingly, an extra increase in photosynthesis was not observed when PSS was further decreased (at PSS 0.70). This could have been due to the decreased stomatal conductance at the lowest PSS value (Table 4). Literature reports different effects of additional FR on photosynthesis, even in one species. For instance, Barreiro et al. (1992) reported for *Phaseolus vulgaris L.* a decrease in photosynthesis with increasing FR, while Holmes et al. (1986) reported opposite. Barreiro et al. (1992) did not measure stomatal conductance, but their results suggested a decrease in stomatal density due to exposure to additional FR. Holmes et al. (1986) reported positive effects of the addition of FR on stomatal opening (short-term) and stomatal conductance on the long term, but did not measure stomatal density. The different PAR light sources and levels of FR used in different researches make it difficult to compare the results. Present results on tomato suggest a negative effect of the absence of FR on photosynthesis due to the absence of the Emerson enhancement effect, while after growth under further increasing FR stomatal conductance decreases. These effects of FR on photosynthesis warrant further investigation.

### Effects of EOD-FR and Broadband Spectrum

In contrast to continuous FR, EOD-FR did not affect petiole angle in EXP1 and did not result in more upright petioles in EXP2, compared to observations in plants with the highest PSS (Figure 1B). Petiole angles were measured at midday, and this may explain the differences in results observed in the EOD-FR treated plants compared to those in plants under continuous FR. Petiole angle may have only been affected during the 15 min EOD-FR application, returning to its initial state after the application. However, the kinetics of this response was not studied.

Tomato plants grown under continuous FR developed an irregular leaf-lamina orientation unlike the plants grown under only EOD-FR (Supplementary Figure S5). This may be due to the fact that plants grown under continuous FR develop phototropic responses to reflected FR, and forage for light in canopy gaps (Ballare et al., 1990, 1997). Under EOD-FR or total absence of FR, the sensitivity of neighbor detection was reduced, and a different phototropic leaf orientation was found, with leaves oriented toward the LED lamps containing blue wavelengths.

In EXP1 all the incident light on the plants was from narrow band LEDs, while in EXP2 the plants also received broad spectrum sunlight. At midday, the solar light in EXP2 increased the blue photon percentage to approximately 10% (from 5%) and green to approximately 8% (from 0%). The addition of blue photons could have reduced the SAS effects, including the retardation of stem elongation (Lin et al., 1998; Pierik et al., 2009). However, green light may reverse the effects of blue light on plant morphology and may result in a plant morphology similar to that observed in FR-induced SAS (Zhang et al., 2011; Wang and Folta, 2013). It should be noted, however, that the limited above-mentioned spectral changes due to solar light were measured when solar radiation was maximal (around noon), and that the effects on the PSS values at that moment were negligible. Together, this may explain why similar responses to all treatments were observed in EXP1 and EXP2. Nevertheless, plants from all treatments in EXP2 had a higher total height and leaf area than the corresponding treatments in EXP1, due to a higher total incident PAR.

## Conclusion

Increasing the R:FR ratio of artificial LED light above the R:FR ratio value for sun light negatively influences the growth and early fruit production of young tomato plants. The observed reductions in plant dry mass due to a lack of FR were mainly related to reductions in whole plant light absorption, which in turn were largely due to reductions in total leaf area. In contrast to the decreased leaf area, the changes in petiole angle and decreased internode length did not negatively influence whole plant light interception in these experiments. Finally, FR increased fruit yield, which correlated well with the accelerated flowering and overall increase in plant source strength under FR light. We conclude that growing tomato plants under artificial light without FR during the light period causes a range of inverse shade avoidance responses, which result in reduced plant source strength and reduced fruit production that cannot be compensated for by a simple EOD-FR treatment. Consequently, in greenhouse horticulture where often RB LEDs are used without additional FR, the addition of FR can result in increased plant growth and fruit production.

## Author Contributions

PK, WvI, JH, and LM conceived and designed the experiments. PK, SM, and KW performed the experiments. PK, MdvM, SM, and KW performed the statistical analysis. MvdM contributed the analysis tools. CN contributed the materials. PK wrote the manuscript. MvdM wrote sections of the manuscript. All authors contributed to manuscript revision, read and approved the submitted version.

## Conflict of Interest Statement

The authors declare that the research was conducted in the absence of any commercial or financial relationships that could be construed as a potential conflict of interest.
