# Supplementary material for: Effects of Continuous or End-of-Day Far-Red Light on Tomato Plant Growth, Morphology, Light Absorption, and Fruit Production
Source: Front Plant Sci. 2019 Mar 28;10:322. doi: 10.3389/fpls.2019.00322 (PMC6448094; doi:10.3389/fpls.2019.00322)
Supplement: Supplementary file 1 [file Table_1.DOCX]

Supplementary Material

Effects of continuous or end-of-day far-red light on tomato plant growth, morphology, light absorption and fruit production

Pavlos Kalaitzoglou^1^, Wim van Ieperen^1^, Jeremy Harbinson^1^, Maarten van der Meer^1^, Stavros Martinakos^1^, Kees Weerheim^1^, Celine Nicole^2^, Leo. F. M. Marcelis^1, *^

*** Correspondence:** L. F. M. Marcelis: [leo.marcelis@wur.nl](mailto:leo.marcelis@wur.nl)

**Supplementary Figure 1.** Spectral PFD for the five treatments without solar light (EXP1) or with solar light (EXP2). Data was acquired in every plot using a spectroradiometer (USB2000 spectrometer, Ocean Optics, Duiven, The Netherlands) on the 12^th^ of February (9:20-11:20 AM solar time) and on the 17^th^ of March (9:20-10:20 AM solar time) for EXP 1 and 2 respectively.

**Supplementary Figure 2.** 3D mock-up of the experimental setup with virtual plants, re-constructed by architectural measurements at the final harvest. Curtains and LEDs are not shown.

**Supplementary Figure 3.** Simple illustrations of rachis curvature in the functional-structural plant model. In the left picture, a petiole angle of 30° is followed by a rachis curvature of 180° between a1 and a2. Leaflets are horizontally positioned, regardless of rachis curvature. On the right picture a petiole angle of 30° is followed by a rachis curvature of 0°.

**Supplementary Figure 4.** A simple illustration of a leaflet curvature of 0°, 15° and 30° in the functional-structural plant model. The base of the leaflet is always horizontal.

**Supplementary Figure 5.** Tomato leaf orientation in relation to four levels of PSS and an EOD FR treatment in EXP1. Plants had been grown for four weeks at the different light treatments.

**Supplementary Table 1.** Concentration of macro- and micro-nutrients inside the nutrient solution.

| **Nutrient** | **mmol l^-1^** | **micromole l^-1^** |
| --- | --- | --- |
| **NH4** | 1.2 |  |
| **K** | 7.2 |  |
| **Ca** | 4.0 |  |
| **Mg** | 1.82 |  |
| **NO3** | 12.4 |  |
| **SO4** | 3.3 |  |
| **P** | 1.0 |  |
| **Fe** |  | 35.0 |
| **Mn** |  | 8.0 |
| **Zn** |  | 5.0 |
| **B** |  | 20.0 |
| **Cu** |  | 0.5 |
| **Mo** |  | 0.5 |

**Supplementary Table 2.** Temperature, relative humidity (RH) and CO_2_ concentration during the growth of tomato plants under LED (EXP1) or LED and solar light (EXP2). The growth period includes germination, light treatment phase and extended experimental phase for EXP2.

|  |  | **EXP1 (LED)** |  | **EXP2 (LED + solar)** |
| --- | --- | --- | --- | --- |
| **Germination Temperature (°C)** |  | 22 |  | 19.5 |
| **Germination RH (%)** |  | 64 |  | 61 |
| **Treatment Day Temperature (°C)** |  | 23 |  | 23 |
| **Treatment Night Temperature (°C)** |  | 18 |  | 18 |
| **Treatment RH (%)** |  | 56 |  | 56 |
| **Treatment CO_2_ (ppm)** |  | 437 |  | 418 |
| **Extended period Temperature (°C)** |  | - |  | 22.5 |
| **Extended period RH (%)** |  | - |  | 64 |
| **Extended period CO_2_ (ppm)** |  | - |  | 394 |

**Supplementary Table 3.** Area and length of different organs in relation to four levels of PSS and an EOD FR treatment, when grown under LED (EXP1) or LED and solar light (EXP2). Different letters indicate significantly different means (*P*<0.05).

|  |  | **EXP1 (LED)** | | | | |  | **EXP2 (LED + solar)** | | | | |
| --- | --- | --- | --- | --- | --- | --- | --- | --- | --- | --- | --- | --- |
| **PSS** |  | **0.70** | **0.73** | **0.80** | **0.88** | **EOD** |  | **0.70** | **0.73** | **0.80** | **0.87** | **EOD** |
| **Leaf 2 (cm^2^)** |  | 288 b | 292 b | 275 ab | 223 a | 233 a |  | 297 b | 273 b | 276 b | 180 a | 293 b |
| **Leaf 3 (cm^2^)** |  | 389 a | 429 b | 439 b | 362 a | 364 a |  | 517 b | 476 ab | 573 b | 457 a | 478 ab |
| **Leaf width (cm)** |  | 32.30 b | 31.65 b | 34.10 b | 27.42 a | 28.06 a |  | 37.57 c | 37.42 c | 35.43 ab | 34.37 a | 36.45 bc |
| **Leaf length (cm)** |  | 24.24 b | 23.84 b | 23.83 b | 21.83 a | 22.21 a |  | 25.91 c | 25.67 bc | 25.11 ab | 24.86 a | 25.98 c |
| **Petiole 2 (cm)** |  | 7.64 ab | 6.99 a | 7.95 ab | 8.93 b | 9.52 b |  | 7.81 a | 7.91 ab | 8.83 bc | 9.05 c | 7.92 ab |
| **Petiole 3 (cm)** |  | 8.43 a | 8.02 a | 8.87 ab | 8.67 ab | 9.50 b |  | 7.15 a | 8.03 b | 8.16 b | 8.33 b | 8.14 b |
| **Petiole (cm)** |  | 6.92 b | 6.77 a | 7.26 b | 6.68 a | 6.69 a |  | 6.82 a | 7.04 b | 7.18 c | 7.26 c | 6.84 a |
| **Internode 2 (cm)** |  | 8.55 d | 8.01 d | 5.63 b | 4.41 a | 7.01 c |  | 11.71 e | 10.13 d | 6.74 b | 5.10 a | 7.69 c |
| **Internode 3 (cm)** |  | 12.09 c | 12.45 c | 9.08 b | 5.71 a | 8,84 b |  | 14.13 d | 13.36 d | 10.12 c | 6.52 a | 9.12 b |
| **Hypocotyl (cm)** |  | 2.78 c | 2.52 c | 1.44 ab | 1.33 a | 1.75 a |  | 1.83 c | 1.42 b | 0.95 a | 1.19 ab | 1.34 b |

**Supplementary Table 4.** Dry weight of tomato plants in relation to four levels of PSS and an EOD FR treatment, when grown under LED (EXP1) or LED and solar light (EXP2). Different letters indicate significantly different means (*P*<0.05).

|  |  | **EXP1 (LED)** | | | | |  | **EXP2 (LED + solar)** | | | | |
| --- | --- | --- | --- | --- | --- | --- | --- | --- | --- | --- | --- | --- |
| **PSS** |  | **0.70** | **0.73** | **0.80** | **0.88** | **EOD** |  | **0.70** | **0.73** | **0.80** | **0.87** | **EOD** |
| **DW leaf 2 (g)** |  | 0.68 ab | 0.59 ab | 0.63 ab | 0.75 b | 0.43 a |  | 1.00 b | 0.89 a | 0.86 a | 0.85 a | 0.90 a |
| **DW leaf 3 (g)** |  | 0.85 a | 0.86 ab | 0.88 ab | 1.01 ab | 0.82 a |  | 1.29 ab | 1.25 a | 1.40 b | 1.36 ab | 1.36 ab |
| **DW all leaves (g)** |  | 8.63 b | 8.38 b | 8.97 b | 8.69 b | 7.55 a |  | 13.23 a | 13.95 b | 14.13 b | 14.18 b | 13.42 ab |
| **LMA (g cm^-2^)** |  | 23.2 a | 22.8 a | 24.1 a | 30.6 c | 26.1 b |  | 26.5 a | 29.8 b | 28.7 ab | 34.5 c | 30.4 b |
| **DW hypocotyl (g)** |  | 0.15 c | 0.14 c | 0.09 b | 0.07 a | 0.08 ab |  | 0.09 c | 0.08 b | 0.07 bc | 0.06 a | 0.07 b |
| **DW internode 2 (g)** |  | 0.50 c | 0.50 c | 0.36 b | 0.24 a | 0.33 b |  | 0.65 c | 0.62 c | 0.46 b | 0.34 a | 0.44 b |
| **DW internode 3 (g)** |  | 0.77 d | 0.74 d | 0.60 c | 0.33 a | 0.45 b |  | 0.95 d | 0.92 d | 0.75 c | 0.47 a | 0.57 b |
| **DW root (g)** |  | 2.12 b | 1.69 ab | 1.87 b | 1.38 a | 1.57 ab |  | 1.54 c | 1.40 b | 1.37 b | 1.33 ab | 1.21 a |
| **DW stem (g)** |  | 6.88 d | 6.50 d | 5.83 c | 2.48 a | 3.70 b |  | 8.81 d | 8.59 d | 7.65 c | 4.57 a | 5.66 b |
