# Supplementary figures and images for: Effects of Continuous or End-of-Day Far-Red Light on Tomato Plant Growth, Morphology, Light Absorption, and Fruit Production
Source: Front Plant Sci. 2019 Mar 28;10:322. doi: 10.3389/fpls.2019.00322 (PMC6448094; doi:10.3389/fpls.2019.00322)

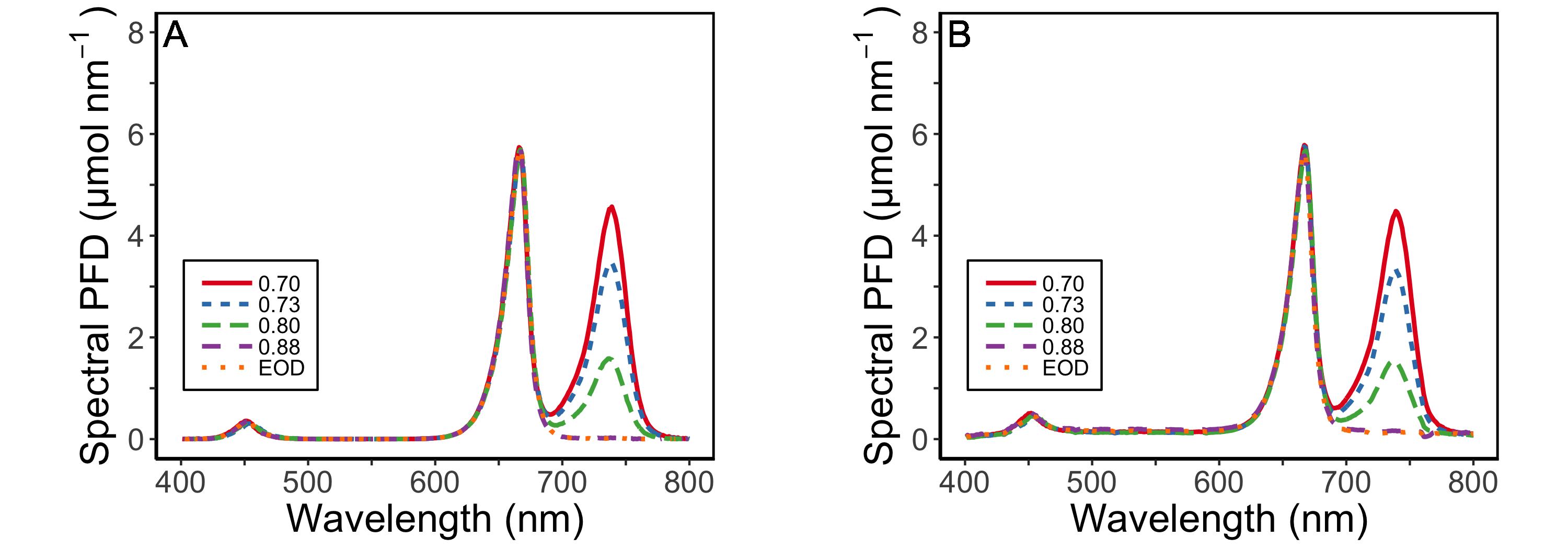

Supplement: Supplementary file 2 [file Image_1.TIFF]

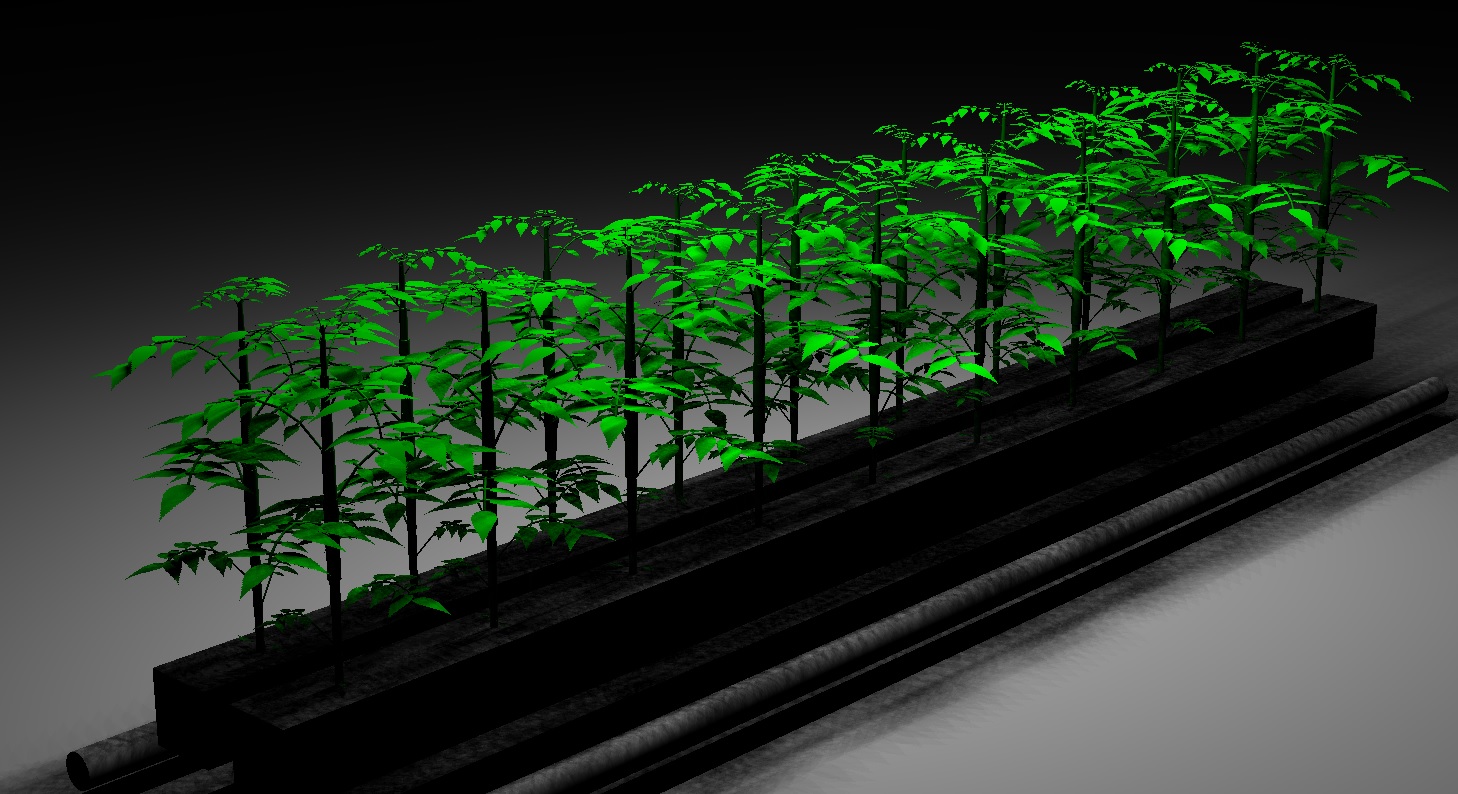

Supplement: Supplementary file 3 [file Image_2.JPEG]

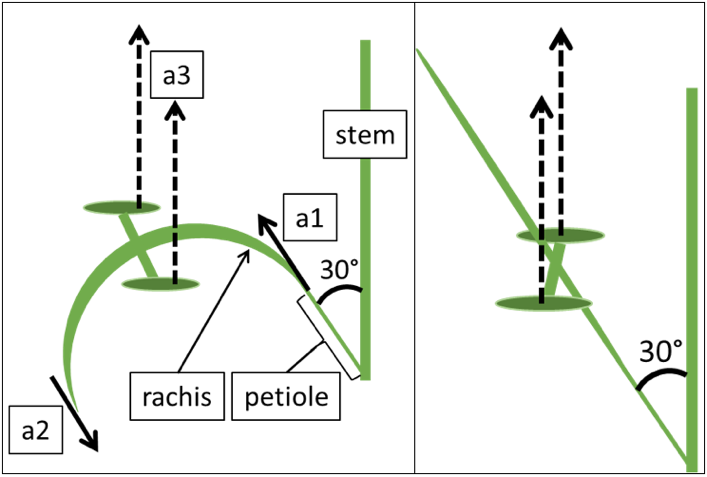

Supplement: Supplementary file 4 [file Image_3.TIFF]

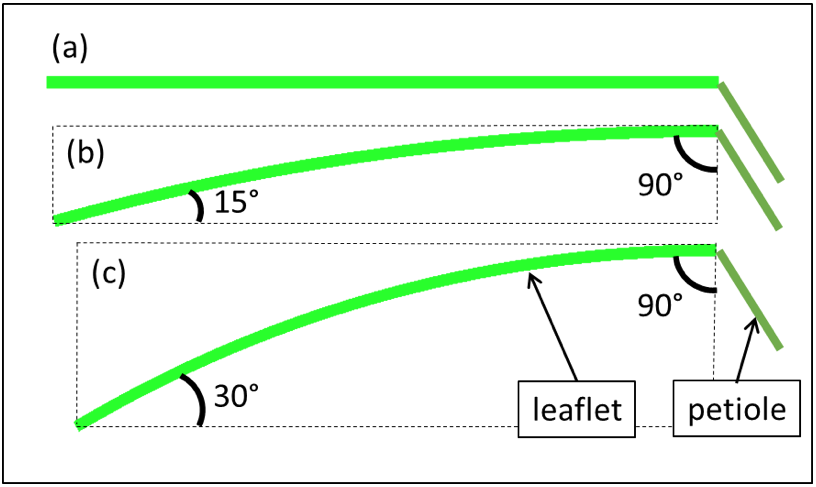

Supplement: Supplementary file 5 [file Image_4.TIFF]

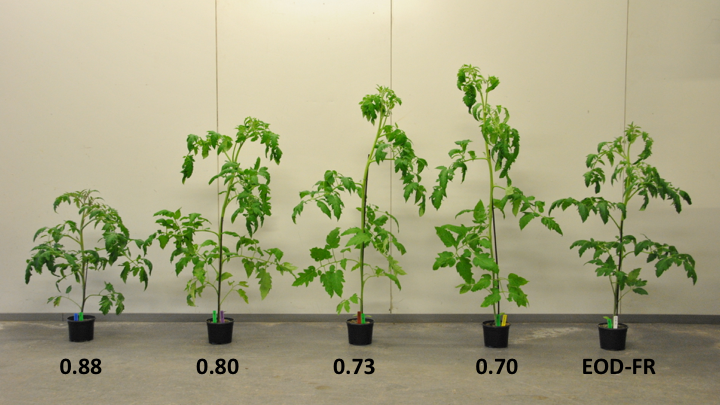

Supplement: Supplementary file 6 [file Image_5.TIFF]
